# Comparing Cognitive Function Tools and Their Relationships with Physical Frailty Measures

**DOI:** 10.1093/geroni/igaf122.2365

**Published:** 2025-12-31

**Authors:** Abigail Tice, Janet Lopez, Dahee Kim, Rui Xie, Ladda Thiamwong

**Affiliations:** University of Central Florida, Orlando, Florida, United States; University of Central Florida, Orlando, Florida, United States; University of Central Florida, Orlando, Florida, United States; University of Central Florida, Orlando, Florida, United States; University of Central Florida, Orlando, Florida, United States

## Abstract

Declining cognitive function and physical frailty can independently decrease quality of life and increase dependence and mortality in older adults. While previous works demonstrate relationships between physical and cognitive frailty, studies investigating differences between cognitive function tools and their associations with physical frailty in older adults are lacking. This study aimed to investigate the relationship between various cognitive tools and physical frailty markers in community-dwelling older adults residing in low-income settings. Older adults (≥60 years old) living in the community were recruited (n = 118). Participants completed cognitive functioning assessments, including, Digital Symbol Substitution Test (DSST), Trail Making Test (TMT)-A, TMT-B, Rowland University Dementia Assessment Scale (RUDAS), and Memory Impairment Screen (MIS). Physical frailty was assessed using the Fatigue, Resistance, Ambulatory, Illness, and Loss of weight (FRAIL) questionnaire, short physical performance battery (SPPB), BTracks Balance system, timed-up-and-go (TUG), 30-second sit-to-stand (STS), and handgrip strength (HGS). Spearman correlations were performed (p < 0.05) using GraphPad Prism. FRAIL score was significantly correlated with TMT-A, TMT-B, and DSST. SPPB total was significantly correlated with all cognitive tests. SPPB balance was significantly correlated with RUDAS and MIS, gait speed with TMT-A, TMT-B, and DSST, and chair stand with RUDAS, MIS, TMT-A, and TMT-B. STS was significantly correlated with RUDAS, TMT-A, TMT-B, and DSST. TUG was significantly correlated with TMT-A, TMT-B, and DSST. HGS was significantly correlated with TMT-A and DSST. Findings suggest that tools measuring different aspects of cognitive function have varying relationships with physical frailty. Future work should be done to dissect these relationships better.

